# Supplementary material for: Association between non-malignant monoclonal gammopathy and adverse outcomes in chronic kidney disease: A cohort study
Source: PLoS Med. 2020 Feb 28;17(2):e1003050. doi: 10.1371/journal.pmed.1003050 (PMC7048272; doi:10.1371/journal.pmed.1003050)
Supplement: S1 Protocol — (DOCX) [file pmed.1003050.s002.docx]

**S1 Protocol:** The prospectively written analysis plan for the study.

# MGUS as a prognostic factor in CKD

### Introduction

Monoclonal gammopathy of undetermined significance (MGUS), defined as the presence of a monoclonal protein (paraprotein) without paraprotein-related end organ damage, is associated with a higher risk of mortality, due at least in part to evolution of the gammopathy into malignant disease [1-3]. It is also known that in some patients who would previously have been diagnosed as having MGUS, the paraprotein is causally associated with kidney damage, now termed monoclonal gammopathy of renal significance (MGRS) [4].

Many patients with chronic kidney disease (CKD) have MGUS. It has been estimated that approximately 35% of patients with MGUS have chronic kidney disease (CKD), suggesting that there is a higher prevalence in this population compared to the general population [5, 6].

Among patients with CKD, the prognostic significance of the presence of MGUS is not known. Only one study, the Chronic Renal Impairment in Birmingham (CRIB) study, has published data on this to date [7]. In this cohort of 364 patients, of whom 35 had MGUS, the presence of MGUS was not independently associated with risk of either ESRD or mortality. However, the associations merit further assessment in a larger cohort of patients with more events.

This work will aim to assess whether the presence of MGUS is a prognostic factor in CKD by amalgamating data from four prospective CKD cohort studies. Two outcomes will be assessed: i) survival, and ii) progression to ESRD.

### Methods

*Study population*

- Data will be pooled from three prospective CKD cohort studies: CRISIS [8], RRID [9], and RIISC [10]. This should provide a cohort of over 3500 patients:

| Cohort | CKD stage, population | Period of recruitment | No. of participants | No. with paraprotein |
| --- | --- | --- | --- | --- |
| CRISIS | 3-5, secondary care | 2004-2010 | 919 | 47 |
| RRID | 3, primary care | 2008-2010 | 1741 | 46 |
| RIISC | 3-5, secondary care | 2010-2015 | 931 | ? |

- Exclusion criteria: patients with non-MGUS monoclonal disease will be excluded (i.e. multiple myeloma, AL amyloidosis, lympho-proliferative diseases). All other patients will be included in the analysis.

*Data*

- Main predictor variable: MGUS status as a binary measure, defined as the presence of a serum monoclonal protein at a concentration < 30 g/L, and/or the presence of an abnormal sFLC ratio (using the renal reference range, 0.37-3.1 [11]) with an increased level of the appropriate involved light chain.
- Main outcome variables:
  1. Progression to ESRD, defined as the initiation of renal replacement therapy
  2. Death
- Other variables in the dataset will comprise all routinely collected clinical and laboratory data associated with the primary outcomes:
  1. Demographic: age, sex, ethnicity
  2. Clinical: primary renal disease, co-morbidities (including cardiovascular and diabetes mellitus), blood pressure, smoking history
  3. Biochemical: creatinine, urine ACR or PCR, calcium, phosphate, albumin, bicarbonate, haemoglobin, CRP
- The full dataset is shown in appendix.

### *Summary of analysis plan*

1. Amalgamation of the three datasets, data tidying where needed, and assessment of the degree of missing data.
2. Exclusion of patients with non-MGUS monoclonal gammopathy.
3. Descriptive baseline characteristics for the whole cohort and by MGUS status.
4. Comparison of baseline factors by MGUS status (t-test for normally-distributed variables, Mann–Whitney U test for non-normally-distributed variables, chi-squared test for categorical variables).
5. Competing-risks regression (Fine and Gray [12]) to determine the association of MGUS status with risk of ESRD and death:
   1. Univariable (adjusted only for study of origin).
   2. Multivariable, adjusting for study of origin, and important confounding prognostic factors such as age, sex, eGFR, ACR, blood pressure.

### References

1. Rajkumar SV, Dimopoulos MA, Palumbo A, Blade J, Merlini G, Mateos MV, et al. International Myeloma Working Group updated criteria for the diagnosis of multiple myeloma. Lancet Oncol. 2014;15(12):e538-48. doi: 10.1016/S1470-2045(14)70442-5. PubMed PMID: 25439696.

2. Kyle RA, Therneau TM, Rajkumar SV, Larson DR, Plevak MF, Melton LJ, 3rd. Long-term follow-up of 241 patients with monoclonal gammopathy of undetermined significance: the original Mayo Clinic series 25 years later. Mayo Clin Proc. 2004;79(7):859-66. doi: 10.1016/S0025-6196(11)62151-4. PubMed PMID: 15244381.

3. Kristinsson SY, Bjorkholm M, Andersson TM, Eloranta S, Dickman PW, Goldin LR, et al. Patterns of survival and causes of death following a diagnosis of monoclonal gammopathy of undetermined significance: a population-based study. Haematologica. 2009;94(12):1714-20. doi: 10.3324/haematol.2009.010066. PubMed PMID: 19608666; PubMed Central PMCID: PMCPMC2791946.

4. Leung N, Bridoux F, Hutchison CA, Nasr SH, Cockwell P, Fermand JP, et al. Monoclonal gammopathy of renal significance: when MGUS is no longer undetermined or insignificant. Blood. 2012;120(22):4292-5. doi: 10.1182/blood-2012-07-445304. PubMed PMID: 23047823.

5. Šálek T, Moravčíková D, Humpolíček P, Tichý M, Palička V. The prevalence of decreased glomerular filtration rate in patients with monoclonal gammopathy of undetermined significance. Klinická biochemie a metabolismus : časopis České společnosti klinické biochemie. 2013;21(1):25-9.

6. Stelmach-Goldys A, Czarkowska-Paczek B, Wyczalkowska-Tomasik A, Paczek L. Serum cystatin C and serum and urine NGAL in the kidney function assessment of patients with MGUS. Eur J Haematol. 2015;94(2):162-8. doi: 10.1111/ejh.12413. PubMed PMID: 25046079.

7. Haynes R, Hutchison CA, Emberson J, Dasgupta T, Wheeler DC, Townend JN, et al. Serum free light chains and the risk of ESRD and death in CKD. Clin J Am Soc Nephrol. 2011;6(12):2829-37. doi: 10.2215/CJN.03350411. PubMed PMID: 22034503; PubMed Central PMCID: PMCPMC3255360.

8. Ritchie J, Assi LK, Burmeister A, Hoefield R, Cockwell P, Kalra PA. Association of Serum Ig Free Light Chains with Mortality and ESRD among Patients with Nondialysis-Dependent CKD. Clin J Am Soc Nephrol. 2015;10(5):740-9. doi: 10.2215/CJN.09660914. PubMed PMID: 25825483; PubMed Central PMCID: PMCPMC4422245.

9. Assi LK, McIntyre N, Fraser S, Harris S, Hutchison CA, McIntyre CW, et al. The Association between Polyclonal Combined Serum Free Light Chain Concentration and Mortality in Individuals with Early Chronic Kidney Disease. PLoS One. 2015;10(7):e0129980. doi: 10.1371/journal.pone.0129980. PubMed PMID: 26132658; PubMed Central PMCID: PMCPMC4489104.

10. Stringer S, Sharma P, Dutton M, Jesky M, Ng K, Kaur O, et al. The natural history of, and risk factors for, progressive chronic kidney disease (CKD): the Renal Impairment in Secondary care (RIISC) study; rationale and protocol. BMC Nephrol. 2013;14:95. doi: 10.1186/1471-2369-14-95. PubMed PMID: 23617441; PubMed Central PMCID: PMCPMC3664075.

11. Hutchison CA, Harding S, Hewins P, Mead GP, Townsend J, Bradwell AR, et al. Quantitative assessment of serum and urinary polyclonal free light chains in patients with chronic kidney disease. Clin J Am Soc Nephrol. 2008;3(6):1684-90. doi: 10.2215/CJN.02290508. PubMed PMID: 18945993; PubMed Central PMCID: PMCPMC2572283.

12. Fine JP, Gray RJ. A Proportional Hazards Model for the Subdistribution of a Competing Risk. Journal of the American Statistical Association. 1999;94(446):496-509. doi: 10.2307/2670170.

**Appendix: Variables required for full dataset**

Baseline variables:

1. Age (or date of birth)
2. Sex
3. Ethnicity
4. Smoking status
5. Co-morbidities
   1. Non-MGUS monoclonal disease:
      1. Multiple myeloma
      2. AL amyloidosis
      3. Other lymphoproliferative disease
   2. Diabetes Mellitus
   3. Ischaemic heart disease
   4. Cerebrovascular disease
   5. Peripheral vascular disease
   6. Malignancy
6. Primary renal disease
7. Blood pressure
8. Laboratory
   1. Creatinine
   2. eGFR
   3. ACR or PCR
   4. Haemoglobin
   5. Bicarbonate
   6. Calcium
   7. Phosphate
   8. Serum albumin
   9. CRP
9. MGUS status
   1. MGUS (yes/no)
   2. Serum FLC – kappa, lambda, combined
   3. Paraprotein type (immunofixation)
   4. Paraprotein quantification (protein electrophoresis)

Outcome and time-to-event data

1. Date of baseline visit
2. ESRD (yes/no) and date
3. Death (yes/no) and date
   - 1. Cause of death
4. Date of end of observation period, if not reached end-point
