# Supplementary material for: Association between non-malignant monoclonal gammopathy and adverse outcomes in chronic kidney disease: A cohort study
Source: PLoS Med. 2020 Feb 28;17(2):e1003050. doi: 10.1371/journal.pmed.1003050 (PMC7048272; doi:10.1371/journal.pmed.1003050)
Supplement: S1 Table — Continuous variables are linear per 1 standard deviation unless otherwise indicated. Transformed continuous variables are indicated by the following footnotes: (a) x3; (b) x−2; (c) x-2ln(x); (d) ln(x); (e) x−1; (f) x0.5. ACR, albumin-to-creatinine ratio; CI, confidence interval; CKD, chronic kidney disease; COPD, chronic obstructive pulmonary disease; DM, diabetes mellitus; eGFR, estimated glomerular filtration rate; HR, hazard ratio; IHD, ischaemic heart disease; MAP, mean arterial pressure; MG, monoclonal gammopathy; PAD, peripheral artery disease; RIISC, Renal Impairment in Secondary Care; SHR, subhazard ratio. (DOCX) [file pmed.1003050.s003.docx]

**S1 Table:** Complete case analyses of the association between baseline variables and risk of kidney failure (competing risks regression, expressed as SHR with 95% CI) and death (Cox proportional hazards regression, expressed as HR with 95% CI) in 878 participants from the RIISC study with CKD. Continuous variables are linear per one standard deviation unless otherwise indicated. Transformed continuous variables are indicated by: a = *x*^3^; b = *x*^-2^; c = *x*^-2^ln(*x*); d = ln(*x*); e = *x*^-1^; f = *x*^0.5^.

ACR albumin-to-creatinine ratio; CI confidence interval; CKD chronic kidney disease; COPD chronic obstructive pulmonary disease; DM diabetes mellitus; eGFR estimated glomerular filtration rate; HR hazard ratio; IHD ischaemic heart disease; MAP mean arterial pressure; MG monoclonal gammopathy; PAD peripheral artery disease; RIISC renal impairment in secondary care; SHR subhazard ratio.

| **Variable** | **Kidney failure** | | | | | | **Death** | | | | | |
| --- | --- | --- | --- | --- | --- | --- | --- | --- | --- | --- | --- | --- |
|  | **Univariable** | | | **Multivariable** | | | **Univariable** | | | **Multivariable** | | |
|  | **SHR** | **95% CI** | ***P*** | **SHR** | **95% CI** | ***P*** | **HR** | **95% CI** | ***P*** | **HR** | **95% CI** | ***P*** |
| **MG+** | 0.97 | 0.68 to 1.36 | 0.84 | 0.92 | 0.60 to 1.40 | 0.69 | 2.13 | 1.49 to 3.04 | <0.001 | 1.29 | 0.86 to 1.93 | 0.22 |
| **Age** | 1.00^a^ | 1.00 to 1.00 | <0.001 | 1.00^a^ | 0.99 to 1.00 | <0.001 | 3.36 | 2.73 to 4.14 | <0.001 | 2.80 | 2.15 to 3.64 | <0.001 |
| **Male sex** | 0.99 | 0.79 to 1.24 | 0.92 | 0.52 | 0.40 to 0.68 | <0.001 | 1.27 | 0.95 to 1.69 | 0.11 | 0.88 | 0.62 to 1.23 | 0.45 |
| **Ethnicity** |  |  |  |  |  |  |  |  |  |  |  |  |
| White | Ref |  |  | Ref |  |  | Ref |  |  | Ref |  |  |
| Asian | 2.02 | 1.57 to 2.58 | <0.001 | 1.27 | 0.95 to 1.71 | 0.11 | 0.51 | 0.33 to 0.79 | 0.002 | 0.99 | 0.61 to 1.60 | 0.97 |
| Black | 1.98 | 1.42 to 2.76 | <0.001 | 1.73 | 1.18 to 2.52 | 0.005 | 0.80 | 0.48 to 1.33 | 0.39 | 1.02 | 0.58 to 1.79 | 0.95 |
| Other | 2.64 | 1.09 to 6.43 | 0.032 | 1.80 | 0.56 to 5.80 | 0.33 | 0.56 | 0.08 to 4.02 | 0.57 | 0.60 | 0.08 to 4.61 | 0.63 |
| **Co-morbidities** |  |  |  |  |  |  |  |  |  |  |  |  |
| DM | 0.92 | 0.73 to 1.15 | 0.46 |  |  |  | 1.64 | 1.25 to 2.17 | <0.001 | 1.40 | 1.03 to 1.91 | 0.033 |
| IHD | 0.85 | 0.65 to 1.10 | 0.22 |  |  |  | 2.44 | 1.83 to 3.24 | <0.001 | 1.35 | 0.98 to 1.86 | 0.06 |
| Cerebrovascular disease | 0.77 | 0.53 to 1.12 | 0.17 |  |  |  | 1.97 | 1.38 to 2.80 | <0.001 | 1.22 | 0.83 to 1.80 | 0.32 |
| PAD | 0.86 | 0.60 to 1.25 | 0.44 |  |  |  | 2.21 | 1.55 to 3.15 | <0.001 | 1.27 | 0.84 to 1.92 | 0.26 |
| COPD | 0.45 | 0.28 to 0.72 | 0.001 |  |  |  | 1.46 | 1.00 to 2.15 | 0.05 | 1.19 | 0.79 to 1.81 | 0.41 |
| Malignancy | 0.51 | 0.35 to 0.75 | 0.001 |  |  |  | 2.16 | 1.57 to 2.96 | <0.001 | 1.46 | 1.02 to 2.09 | 0.040 |
| **Smoking status** |  |  |  |  |  |  |  |  |  |  |  |  |
| Never | Ref |  |  |  |  |  | Ref |  |  | Ref |  |  |
| Previous | 0.69 | 0.54 to 0.88 | 0.003 |  |  |  | 1.73 | 1.28 to 2.34 | <0.001 | 1.08 | 0.76 to 1.53 | 0.67 |
| Current | 1.07 | 0.78 to 1.47 | 0.68 |  |  |  | 1.14 | 0.71 to 1.83 | 0.59 | 1.30 | 0.73 to 2.29 | 0.37 |
| **Cause of CKD** |  |  |  |  |  |  |  |  |  |  |  |  |
| Vascular | Ref |  |  | Ref |  |  | Ref |  |  |  |  |  |
| Diabetes | 1.89 | 1.34 to 2.68 | <0.001 | 0.86 | 0.56 to 1.30 | 0.46 | 0.81 | 0.52 to 1.26 | 0.35 |  |  |  |
| Glomerular | 1.18 | 0.80 to 1.74 | 0.39 | 0.97 | 0.62 to 1.51 | 0.88 | 0.22 | 0.11 to 0.42 | <0.001 |  |  |  |
| Tubulointerstitial | 0.90 | 0.57 to 1.41 | 0.65 | 0.51 | 0.30 to 0.85 | 0.010 | 0.31 | 0.16 to 0.58 | <0.001 |  |  |  |
| Cystic or congenital | 2.83 | 1.95 to 4.11 | <0.001 | 3.89 | 2.57 to 5.90 | <0.001 | 0.26 | 0.10 to 0.63 | 0.003 |  |  |  |
| Other or unknown | 1.25 | 0.89 to 1.74 | 0.20 | 1.08 | 0.75 to 1.57 | 0.68 | 0.82 | 0.57 to 1.17 | 0.27 |  |  |  |
| **MAP** | 1.39 | 1.25 to 1.56 | <0.001 | 0.89 | 0.78 to 1.03 | 0.11 | 0.79 | 0.68 to 0.92 | 0.003 |  |  |  |
| **eGFR** | 1.18^b^ | 1.14 to 1.22 | <0.001 | 0.94^b^ | 0.93 to 0.96 | <0.001 | 0.45 | 0.36 to 0.57 | <0.001 | 0.61 | 0.46 to 0.81 | 0.001 |
|  | 1.06^c^ | 1.04 to 1.07 | <0.001 | 3.43^e^ | 2.82 to 4.17 | <0.001 |  |  |  |  |  |  |
| **Urine ACR** | 1.42^d^ | 1.32 to 1.54 | <0.001 | 3.19^f^ | 2.55 to 4.00 | <0.001 | 0.85 | 0.68 to 1.06 | 0.16 | 1.28 | 1.05 to 1.56 | 0.016 |
|  | 1.00^a^ | 1.00 to 1.00 | <0.001 |  |  |  |  |  |  |  |  |  |
