# Supplementary material for: Association between non-malignant monoclonal gammopathy and adverse outcomes in chronic kidney disease: A cohort study
Source: PLoS Med. 2020 Feb 28;17(2):e1003050. doi: 10.1371/journal.pmed.1003050 (PMC7048272; doi:10.1371/journal.pmed.1003050)
Supplement: S2 Table — Continuous variables are linear per 1 standard deviation unless otherwise indicated. Transformed continuous variables are indicated by the following footnotes: (a) x3; (b) x-2; (c) x−2ln(x); (d) x0.5; (e) x2; (f) x−1; (g) ln(x); (h) [ln(x)]2; (i) x0.5ln(x). ACR, albumin-to-creatinine ratio; CI, confidence interval; CKD, chronic kidney disease; DM, diabetes mellitus; eGFR, estimated glomerular filtration rate; HR, hazard ratio; IHD, ischaemic heart disease; LC-MG, light chain monoclonal gammopathy; MAP, mean arterial pressure; PAD, peripheral artery disease; SHR, subhazard ratio. (DOCX) [file pmed.1003050.s004.docx]

**S2 Table:** Complete case analyses of the association between baseline variables and risk of kidney failure (competing risks regression, expressed as SHR with 95% CI) and death (Cox proportional hazards regression, expressed as HR with 95% CI) in 3478 participants with CKD from three cohort studies. Continuous variables are linear per one standard deviation unless otherwise indicated. Transformed continuous variables are indicated by: a = *x*^3^; b = *x*^-2^; c = *x*^-2^ln(*x*); d = *x*^0.5^; e = *x*^2^; f = *x*^-1^; g = ln(*x*); h = {ln(*x*)}^2^; i = *x*^0.5^ln(*x*).

ACR albumin-to-creatinine ratio; CI confidence interval; CKD chronic kidney disease; DM diabetes mellitus; eGFR estimated glomerular filtration rate; HR hazard ratio; IHD ischaemic heart disease; LC-MG light chain monoclonal gammopathy; MAP mean arterial pressure; PAD peripheral artery disease; SHR subhazard ratio.

| **Variable** | **Kidney failure** | | | | | | **Death** | | | | | |
| --- | --- | --- | --- | --- | --- | --- | --- | --- | --- | --- | --- | --- |
|  | **Univariable** | | | **Multivariable** | | | **Univariable** | | | **Multivariable** | | |
|  | **SHR** | **95% CI** | ***P*** | **SHR** | **95% CI** | ***P*** | **HR** | **95% CI** | ***P*** | **HR** | **95% CI** | ***P*** |
| **LC-MG+** | 1.07 | 0.57 to 2.01 | 0.83 | 1.43 | 0.73 to 2.79 | 0.30 | 2.51 | 1.58 to 3.97 | <0.001 | 1.44 | 0.88 to 2.35 | 0.15 |
| **Age** | 1.00^a^ | 1.00 to 1.00 | <0.001 | 1.00^a^ | 1.00 to 1.00 | <0.001 | 2.88 | 2.60 to 3.19 | <0.001 | 2.75 | 2.46 to 3.08 | <0.001 |
| **Male sex** | 0.95 | 0.80 to 1.12 | 0.55 | 1.10 | 0.92 to 1.33 | 0.30 | 1.59 | 1.37 to 1.84 | <0.001 | 1.27 | 1.08 to 1.49 | 0.004 |
| **Ethnicity** |  |  |  |  |  |  |  |  |  |  |  |  |
| White | Ref |  |  | Ref |  |  | Ref |  |  | Ref |  |  |
| Asian | 1.94 | 1.54 to 2.44 | <0.001 | 1.02 | 0.79 to 1.33 | 0.85 | 0.67 | 0.47 to 0.94 | 0.022 | 1.24 | 0.86 to 1.78 | 0.26 |
| Black | 1.84 | 1.33 to 2.54 | <0.001 | 1.59 | 1.12 to 2.27 | 0.010 | 0.77 | 0.48 to 1.25 | 0.30 | 1.09 | 0.65 to 1.82 | 0.75 |
| Other | 2.80 | 1.32 to 5.93 | 0.007 | 1.22 | 0.50 to 2.99 | 0.66 | 0.48 | 0.12 to 1.93 | 0.30 | 0.83 | 0.20 to 3.34 | 0.79 |
| **Co-morbidities** |  |  |  |  |  |  |  |  |  |  |  |  |
| DM | 0.92 | 0.77 to 1.09 | 0.33 |  |  |  | 1.71 | 1.47 to 1.97 | <0.001 | 1.41 | 1.21 to 1.65 | <0.001 |
| IHD | 1.03 | 0.83 to 1.27 | 0.80 |  |  |  | 1.64 | 1.37 to 1.96 | <0.001 | 1.31 | 1.10 to 1.55 | 0.003 |
| Cerebrovascular disease | 0.84 | 0.63 to 1.11 | 0.21 |  |  |  | 1.97 | 1.64 to 2.37 | <0.001 | 1.43 | 1.18 to 1.73 | <0.001 |
| PAD | 1.10 | 0.86 to 1.39 | 0.45 |  |  |  | 0.93 | 0.74 to 1.16 | 0.50 | 0.93 | 0.76 to 1.15 | 0.52 |
| **Smoking status** |  |  |  |  |  |  |  |  |  |  |  |  |
| Never | Ref |  |  |  |  |  | Ref |  |  | Ref |  |  |
| Previous | 0.72 | 0.60 to 0.86 | <0.001 |  |  |  | 1.71 | 1.46 to 1.99 | <0.001 | 1.27 | 1.07 to 1.50 | 0.005 |
| Current | 1.14 | 0.90 to 1.46 | 0.28 |  |  |  | 1.33 | 1.03 to 1.73 | 0.030 | 1.54 | 1.16 to 2.04 | 0.003 |
| **MAP** | 1.30 | 1.20 to 1.40 | <0.001 | 1.10 | 1.01 to 1.20 | 0.037 | 0.00^g^ | 0.00 to 0.00 | <0.001 |  |  |  |
|  |  |  |  |  |  |  | 71.39^h^ | 7.59 to 671.83 | <0.001 |  |  |  |
| **eGFR** | 1.17^b^ | 1.15 to 1.20 | <0.001 | 0.96^b^ | 0.95 to 0.97 | <0.001 | 0.00^e^ | 0.00 to 0.01 | <0.001 | 0.64 | 0.56 to 0.72 | <0.001 |
|  | 1.06^c^ | 1.05 to 1.07 | <0.001 | 2.41^f^ | 2.10 to 2.76 | <0.001 |  |  |  |  |  |  |
| **Urine ACR** | 10.86^d^ | 7.13 to 16.52 | <0.001 | 3.56^d^ | 2.65 to 4.76 | <0.001 | 1.70^d^ | 1.23 to 2.37 | 0.002 | 1.14^g^ | 1.07 to 1.21 | <0.001 |
|  | 0.80^e^ | 0.68 to 0.94 | 0.006 |  |  |  | 0.39^i^ | 0.29 to 0.54 | <0.001 | 1.01^h^ | 1.00 to 1.01 | <0.001 |
