# Supplementary material for: Association between non-malignant monoclonal gammopathy and adverse outcomes in chronic kidney disease: A cohort study
Source: PLoS Med. 2020 Feb 28;17(2):e1003050. doi: 10.1371/journal.pmed.1003050 (PMC7048272; doi:10.1371/journal.pmed.1003050)
Supplement: Attachment — Submitted filename: Response_to_reviewers.docx [file pmed.1003050.s005.docx]

19 December 2019

Dr Anthony Fenton

Department of Renal Medicine

Queen Elizabeth Hospital

Mindelsohn Way

Birmingham

B15 2GW

Dr Adya Misra

Senior Editor

PLOS Medicine

Dear Dr Misra,

**Re: Manuscript PMEDICINE-D-19-03271: Association between non-malignant monoclonal gammopathy and adverse outcomes in chronic kidney disease**

We are very grateful for the comments and recommendations from the review of the above manuscript. We have carefully considered these and have amended our manuscript to address them. Below is a summary of the points raised in the review and the changes we have made. Tracked changes in the manuscript are labelled by the editor/reviewer and comment number as indicated in the following list:

**Editor:**

1. *Title: please include a study descriptor to adhere to PLOS Medicine style.*

A study descriptor has now been added. *(p1)*

2. *Abstract: please clarify if a causal association can be inferred from population studies? Please consider toning down or removing.*

We have changed the wording of this part of the abstract. *(p2)*

3. *Abstract: please provide population demographics, ie where the cohorts are from including names of hospitals or countries as appropriate.*

We have now added this information. *(p2)*

4. *Abstract: please introduce FLC ratio on first view.*

The abbreviation and reference to FLC have been removed from the abstract, as part of the simplification recommended in point 7. *(p2)*

5. *Abstract background: please rephrase the first sentence to “In studies including the general population..” or similar for clarity.*

This has been rephrased as recommended. *(p2)*

6. *Abstract methods and findings: please include a limitation of your work as the last sentence of this section.*

We have added a limitation to this section as requested. *(p3)*

7. *Abstract methods section requires clarification. Please simplify the text in the abstract to explain the FLC ratio/LC MG measurement. In line 31-33 you say “Further, to assess the association between a light-chain (LC) MG (defined as an FLC ratio outside the renal reference range with an increased level of the involved light chain) two other cohorts were also studied…”. Could you clarify what association is being referred to here?*

We have removed reference to FLC in the abstract to simplify this section, as it was not felt to be essential to include this information in the abstract. The unclear sentence concerning the association has been removed, as the methods section is clear without it. *(p2)*

8. *Include a short, non-technical Author Summary.*

The has been included. *(p4)*

9. *A. State whether your study had a prospective protocol or analysis plan early in the Methods section. B. Include the document with your revised manuscript as a Supporting Information file to be published alongside your study, and cite it in the Methods section. A legend for this file should be included at the end of your manuscript. C. Changes in the analysis—including those made in response to peer review comments—should be identified as such in the Methods section of the paper, with rationale.*

The prospective analysis plan has been included in supplementary material and cited in the methods. *(p6)*

10. *Indicate: (1) the specific hypotheses you intended to test, (2) the analytical methods by which you planned to test them, (3) the analyses you actually performed, and (4) when reported analyses differ from those that were planned, transparent explanations for differences that affect the reliability of the study’s results.*

Hypotheses and analyses were pre-specified, as indicated in the paper. *(p5)*

Changes in analyses, based on the statistical review, are indicated where appropriate. *(p10)*

11. *Line 100- Please introduce MGRS at first view.*

MGRS is introduced at the first view, in the first paragraph of the introduction. *(p5)*

12. *Please provide p values along with 95% CI where appropriate.*

P values have now been added to all results in the abstracts (already provided everywhere else). *(p2,3)*

13. *Lines 164-165 require clarification- please can you explain what is meant by “more had a history of …” etc?*

These sentences have been rewritten to improve clarity, and now also include the results of statistical tests of differences between those with and those without MG as recommended in the statistical review. The corresponding section for LC-MG has also been rewritten to improve clarity based on these recommendations. *(p10,16)*

14. *Please ensure that the study is reported according to the STROBE guideline, and include the completed STROBE checklist as Supporting Information. Please add the following statement, or similar, to the Methods: "This study is reported as per the Strengthening the Reporting of Observational Studies in Epidemiology (STROBE) guideline (S1 Checklist)." The STROBE guideline can be found here:* [*http://www.equator-network.org/reporting-guidelines/strobe/*](http://www.equator-network.org/reporting-guidelines/strobe/)*. When completing the checklist, please use section and paragraph numbers, rather than page numbers.*

The study is reported according to the STROBE guideline, and this is now stated in the methods section, and the completed checklist is now included in the Supporting Information. *(p6; Supplementary Information)*

15.	*Please present and organize the Discussion as follows: a short, clear summary of the article’s findings; what the study adds to existing research and where and why the results may differ from previous research; strengths and limitations of the study; implications and next steps for research, clinical practice, and/or public policy; one-paragraph conclusion.*

The discussion section has been amended and re-organised to conform to this recommendation. *(p22-24)*

**Reviewer 1:**

1. *First, as they are using data collected for other purposes, adding many variables to the statistical analysis limits the researchers’ ability to determine cause and effect. It is difficult to assess correlation using a retrospective study (RS) design, as RS are subject to numerous threats to validity (limiting interpretation and generalizability of results): the results should be considered as preliminary. Thus, the authors should be very cautious in their conclusions.*

We acknowledge these comments. As such, we have now explicitly stated in the discussion the need for further research and have toned down the wording of our conclusions. *(p23, 24)*

2. *Data collection comes from 3 different sources, with different inclusion criteria, data collected, etc... Define if MG was detected by serum protein electrophoresis, if then patients underwent serum immunosubtraction and/or immunofixation (Immunofixation of a 24 h urine specimen performed to test for Bence Jones protein?). The diagnostic criteria for MGUS also should be included in this article and not referred for readers to the 3 studies used for it (these should all be included in the article itself): it would be great if authors could clarify eGFR formula used (CKD-EPI, MDRD 4...) and include the definition of MG/MGUS. Specify type of Ig related MG if possible.*

Our definitions of MG and LC-MG are given, and we have clarified that serum immunofixation was used to confirm MG after SPEP. *(p8)*

We have clarified that eGFR was calculated using the MDRD formula. *(p9)*

We have added the MG Ig types to the results. *(p10)*

3. *There is a low proportion of events (1.6% including all 3 studies, 55 patients from 3478), which limits statistical power to detect associations: authors should write about their study SUGGESTING X or Y are/aren’t risk factors, but that further prospective studies are necessary to clarify.*

We acknowledge this point and have now added a statement that further research is necessary. (p23)

However, 1.6% simply reflects the prevalence of LC-MG (and is consistent with other estimates of LC-MG prevalence referenced in the discussion). Concerning the number of events (MG analysis: 327 kidney failure and 202 deaths; LC-MG analysis: 564 kidney failure and 803 deaths), which is a more important factor in the power of Cox and Competing Risks regression models, all our multivariable models fall well within the often-stated “10 events per variable” rule.

4. *It would be interesting to know some serologies in these patients, as MG has been related to them (herpes virus, EBV, etc): Babel N, Schwarzmann F, Pruss A, et al. Monoclonal gammopathy of undetermined significance (MGUS) is associated with an increased frequency of Epstein‐Barr Virus (EBV) - Latently infected B lymphocytes in long‐term renal transplant patients. Transplant Proc 2004; 36: 2679; Regamey N, Hess V, Passweg J, et al. Infection with human herpesvirus 8 and transplant‐associated gammopathy. Transplantation 2004; 77: 1551.*

We acknowledge that this would be of interest, but we do not have these data.

5. *Include Monoclonal gammopathy after liver transplantation: a risk factor for long‐term medical complications other than malignancies (DOI 10.1111/j.1432-2277.2011.01362.x), as they refer to CKD and MG (although in liver Tx recipients).*

Thank you for bringing this paper, which presents several interesting findings, to our attention. The paper possibly shows that MGUS after liver transplantation is associated with a higher risk of incident CKD (although without proper adjustment for baseline kidney function). However, as the population (liver transplant recipients) and the question addressed (risk of incident CKD) differ significantly from those of our study, we have respectfully decided against including this paper in our discussion.

6. *Overall, the results indeed suggested that there might be no association between CKD and MG, but no clear cut conclusion should be drawn from this (I would change their strong conclusion phrasing).*

We acknowledge this point and have toned down our concluding sentences. *(p24)*

**Reviewer 2:**

1. *Move the supplemental figures into the text.*

We have now moved the supplementary figures into the text. *(p14,15,19,20,21)*

2. *Line 55-56: This is too strong. This accepts the null. If you want to make this statement, you would have to do tests of equivalence.*

We take this point, and the conclusion of the abstract has been rewritten. *(p3)*

3. *Line 130-131: In general, this is not a reason to not do power analysis. If the authors had done power analysis and found that they needed a larger N, then they would have had to either find more data or do a different analysis. In this specific case, since N is so large, it’s not a problem.*

We accept this point, and we have stated explicitly in the methods that a power analysis was not performed.

4. *Line 137-140 How many extreme points were there? Also, why not use robust Cox regression?*

Rather than truncation of extreme values, we have now re-run all analyses using robust methods for standard error estimation, and all results (including tables and figures) have been updated accordingly. This led to only minor changes in the estimates of the association between MG and adverse outcomes, with no significant change in overall conclusions. *(p10; all results in text, tables, and figures throughout the manuscript)*

5. *Line 141-143 Why was this done? Here, statistical significance is not the issue. Also, the authors don’t seem to use the results of these analyses (unless I missed something)*

When comparing the outcomes between those with MG and those without MG, it is likely to be of interest whether other factors were significantly different at baseline, especially other prognostic factors. These results were used to inform a description of the baseline characteristics that differed, and the *P* values for these analyses have now been included. *(p10,16)*

6. *Line 150 "Stratified" usually means "separate" I’m not sure that this is what is meant here. Was each cohort analyzed separately? It doesn’t look like that was done, from the results. Please clarify. (An alternative would be a multilevel model, if the authors are worried about dependent errors).*

Cohorts were not analysed separately. The use of stratified models on amalgamated data allowed for the estimation of equal coefficients for a predictor across cohorts, but with a baseline hazard unique to each cohort, which numerous methodology papers have recommended as a way of accounting for clustering when amalgamating individual participant data from multiple cohorts. We have changed the wording here for clarification. *(p10)*

*7.	Tables: Why are some sections blank?*

Where variables are not included in a multivariable model, its cells are shaded grey in the column for that particular model. This clarification has now been added to the table descriptions. *(p14,19)*

**Reviewer 3:**

1. *There is no description about amyloidosis. If the authors investigate the association between amyloidosis and the progression of CKD, the results would be useful for nephrologist. Just the definition of MGUS is too wide to consider the role for the progression of CKD.*

The link between AL amyloidosis and progression of CKD is of interest, but our study addresses prognosis in the different, and far more common, clinical scenario of a patient with CKD and non-malignant MG without amyloidosis, which we believe will be of interest to the nephrologist. AL amyloidosis is very rare and develops from MGUS. There is an established literature on the association between AL amyloidosis and kidney disease, and renal outcomes following a diagnosis of AL amyloidosis have been published on.

2. *There is no information on CRP. Since the infection has a key role for the progression of MGUS, the lack of such information should be avoided.*

Unfortunately, CRP data were not available. To our knowledge, there is no substantial body of evidence that demonstrates that infection or an elevated CRP is a significant predictor of MGUS progression.

3. *The data are collected at baseline visits. Although the authors described higher HR of ACR, urinary protein excretion may increase with time. So, it may be inadequate to investigate the role of urinary protein excretion by the baseline value.*

We accept that change in ACR over time would be of interest and may have prognostic significance, but we do not have subsequent urine ACR values for all cohorts to do these analyses. We maintain that our approach is valid, and indeed the incorporation of a single baseline value of ACR, rather than the change in ACR over time, is used in the vast majority of CKD prognostic studies.

**Reviewer 4:**

1. *Do the authors have data to consider other endpoints, such as acute kidney injury, or rapid CKD progression?*

We do not have data on AKI or rapid CKD progression. We have stated as a limitation in our discussion, the lack of analyses of endpoints other than kidney failure and death. *(p24)*

2. *The confidence intervals in the abstract are quite wide, so it is not inconceivable that the study did not have enough power to detect significance at the conventional 5% levels. Could the authors also discuss the possibly of over-adjusting in the multivariable model, for example, adjusting for common confounders between the exposures & the outcomes?*

In the discussion we have stated that it is possible our study was not large enough to detect an association between the presence of an MG and risk of death. (p23-24)

We have reduced the risk of over-adjusting by pre-specifying our multivariable models based on established prognostic factors in CKD (i.e. no data-driven inclusion of variables based on the univariable analyses), and by ensuring that the number of variables included in each multivariable model is well within the ‘10 events per variable’ rule.

3. *Could the authors provide more detail on the process of dealing with the extreme values. Was the truncation process applied to the creatinine measurements as well? Also, am I right that the measurements were not excluded, but rather it is their truncated values were used? How many measurements were truncated in the end?*

Based on comments from the statistical review, to allow for outliers, all analyses have been repeated using robust methods of variance estimation, with no truncation of extreme values. *(p10)*

4. *How was eGFR calculated from creatinine?*

The MDRD formula was used, and this has now been stated in the methods. *(p9)*

5. *Table 1: Inclusion criteria for RIISC: what does "with eGFR decline" mean?*

This has now been added to the table. *(p7)*

Having made the above amendments to the manuscript, we have attached a revised manuscript, and are grateful for your further review and consideration for publication in PLOS Medicine.

Yours sincerely,

Dr Anthony Fenton
