# Supplementary material for: Association between non-malignant monoclonal gammopathy and adverse outcomes in chronic kidney disease: A cohort study
Source: PLoS Med. 2020 Feb 28;17(2):e1003050. doi: 10.1371/journal.pmed.1003050 (PMC7048272; doi:10.1371/journal.pmed.1003050)
Supplement: Attachment — Submitted filename: Response_to_reviewers.docx [file pmed.1003050.s006.docx]

27 January 2020

Dr Anthony Fenton

Department of Renal Medicine

Queen Elizabeth Hospital

Mindelsohn Way

Birmingham

B15 2GW

Dr Adya Misra

Senior Editor

PLOS Medicine

Dear Dr Misra,

**Re: PMEDICINE-D-19-03271R1: Association between non-malignant monoclonal gammopathy and adverse outcomes in chronic kidney disease: a cohort study**

We are very grateful for your further review of the above manuscript. We have addressed the editorial and production issues raised, as summarised below:

**Editor:**

1. *Author summary does not mention that the aim of the study was to compare the rates of MG in the general population with patients who have CKD. Since this is mentioned in the conclusions and discussion through the submission, please clarify if this information should be in the author summary*

This has been now been added to the author summary. *(p4)*

2. *Please use p<0.001 throughout, ensuring p values are provided along with 95% CI*

All *P* values < 0.001 throughout the manuscript have now been changed to this format.

3. *Some summary demographic information would be useful in the abstract*

This information has now been added to the abstract. *(p2)*

4. *Please remove spaces between refs where multiple ones cited*

This has now been done throughout the manuscript.

5. *Data availability- please clarify if the data underlying various graphs and tables can be provided as supplementary information as per PLOS Data policy.*

A restriction enforced by the West Midlands South Birmingham Research Ethics Committee (REC) dictates that data may not be shared. Subject to adherence to existing ethics approval, data may be available on request, and this is stated in the Data Availability statement with the contact details for the REC.

6. *Introduction- Line 83 you say, “MG is a prevalent condition”. It would be helpful to provide context as to its prevalence or a reference for our non-specialist readers*

A reference has been added to this statement, and an estimate of prevalence is included in the next sentence. *(p5)*

**Reviewer 3:**

1. *The authors responded to my comment that "Unfortunately, CRP data were not available. To our knowledge, there is no substantial body of evidence that demonstrates that infection or an elevated CRP is a significant predictor of MGUS progression." But, high CRP is a risk factor of the progression of CKD (Am J Kidney Dis. 2016 ;68(6):873-881, Ren Fail. 2015 ;37(1):45-9.). Since the authors investigated the progression of CKD by non-malignant monoclonal gammopathy, the role of CRP has to be evaluated.*

We agree that it would be of interest to review the association between CRP and progression to kidney failure in our study population and whether there is an interaction with monoclonal gammopathy. However, CRP data were not available. We maintain that our models for risk of kidney failure without CRP are valid, and indeed the vast majority of CKD prognostic studies do not include CRP in their models (including the Tangri et al. Kidney Failure Risk Equation, an equation extensively validated and used most frequently in clinical practice).

**Production:**

1. FIGURES and GRAPHICS:
   a. *Please remove embedded figures from your manuscript file and ensure the most up to date versions of your figures are uploaded to our submission system as separate TIF or EPS files. Please do not remove the figure legends and captions.*

All embedded figures have been removed from the manuscript, and uploaded separately as TIF files.

b. *Please ensure that all main figure files are cited in ascending numerical order in the main text of the article.*

All figure files are cited in ascending numerical order in the text.

c. *Please ensure that all main figures are referenced in the manuscript as Fig 1, Fig 2, etc. (including capitalization), rather than Figure 1, Figure 2, etc.*

All figures are referenced in the manuscript as Fig 1, Fig 2, etc.

d. *Please ensure that all main figure legends are placed after the paragraph containing their first in-text citation.*

All figure legends are placed after the paragraph containing their first citation.

e. *Please note that your figures do not currently comply with our publication requirements.*
   i. *Figures 1-10 are low resolution and will appear blurry when published.*
   ii. *Figures 1, 3, 4, 5, 7, 8, and 9 contain font size that is below our recommended range of font sizes.*

All figures have been redrawn to meet the above specifications.

2. TABLES and BOXES:
   a. *Please cite main tables in ascending numerical order in your main article file.*

All tables are cited in ascending numerical order in the article.

b. *Please ensure that all main tables in their entirety (including titles and footnotes), are placed after the paragraph containing their first in-text citation.*

All tables are placed, in their entirety, after the paragraph containing their first citation.

c. *Table 1 uses returns to control text wrapping.*

These returns have been removed. *(p7)*

3. METADATA:
   a. *Please accurately complete the 'Competing Interests' section on our submission form, including any COIs declared by your co-authors. If you have no competing interests to declare, please add "The authors have declared that no competing interests exist". Otherwise please declare all competing interests beginning with the statement "I have read the journal's policy and the authors of this manuscript have the following competing interests:"*
   b. *Your co-author, Dr. Cockwell, has declared the following competing interest in relation to this manuscript. Please include it in the main competing interest statement in our system, sticking as closely as possible to Dr. Cockwell's wording, using only their initials. "I have consulted for and advised the Binding Site, who are the company who produce the Freelite assay, that has been used to measure the LC MGUS reported in this paper, and who carried out the intact immunoglobulin assays. Harding declared: Board of Directors for The Binding Site who produce an assay used in this submission."*
   c. *Please ensure only the authors' initials are included in the competing interest statement, not their full names or titles.*
   d. *Your co-author, Maarten W. Taal, serves on our Editorial Board. Please include the following sentence as part of your competing interest statement: "[author initials] is a member of the Editorial Board of PLOS Medicine".*

The above competing interests have been added to the Competing Interests section.

e. *In accordance with PLOS' data policy, please ensure that your Data Availability Statement in the submission form clearly identifies how readers can access your data. Data cannot be available on request, or only accessible by contacting one of the authors.*

The data availability statement indicates a restriction enforced by the Research Ethics Committee (REC), and the contact at the REC for data request.

4. ARTICLE FILE:
   a. *Please ensure that your Ethics statement is available in the Methods section of your manuscript in its entirety.*

The full ethics statement has now been added to the methods section. *(p6)*

5. REFERENCES:
   a. *Please ensure your references are in the style of PLOS Medicine (ICMJE/Vancouver style).*

This has been done.

b. *Please put all reference citations in square brackets separated by either commas or dashes with no spaces, e.g. [1,2,3] or [1-3].*

Reference citations have been changed throughout to match this format.

c. *Please make sure all references are cited in ascending numerical order in the text.*

All references are cited in ascending numerical order in the text.

6. SUPPORTING INFORMATION:
   a. *Please ensure that all Supporting Information files use the following format verbatim throughout the manuscript (including legends and all in-text citations): S1 Fig, S1 Table, S1 Text, S1 Data, etc.*
   b. *Please ensure that each supporting information file has a legend at the end of the manuscript file, after the Reference list.*
   c. *Please note that your submission cannot contain any references to generic "supporting information" files and instead must refer to specific files using our naming convention (S1 Fig, S1 Table, S1 Text, S1 Data, SI PRISMA Checklist, etc.)*

All supporting information files and citations to them have been formatted as above.

Yours sincerely,

Dr Anthony Fenton
